# TED-culture: culturally inclusive co-speech gesture generation for embodied social agents

**DOI:** 10.3389/frobt.2025.1546765

**Published:** 2025-04-08

**Authors:** Yixin Shen, Wafa Johal

**Affiliations:** School of Computer Science and IT, FEIT, University of Melbourne, Melbourne, VIC, Australia

**Keywords:** co-speech gesture generation, human-robot interaction, social agents, virtual avatar, humanoid robot

## Abstract

Generating natural and expressive co-speech gestures for conversational virtual agents and social robots is crucial for enhancing their acceptability and usability in real-world contexts. However, this task is complicated by strong cultural and linguistic influences on gesture patterns, exacerbated by the limited availability of cross-cultural co-speech gesture datasets. To address this gap, we introduce the TED-Culture Dataset, a novel dataset derived from TED talks, designed to enable cross-cultural gesture generation based on linguistic cues. We propose a generative model based on the Stable Diffusion architecture, which we evaluate on both the TED-Expressive Dataset and the TED-Culture Dataset. The model is further implemented on the NAO robot to assess real-time performance. Our model surpasses state-of-the-art baselines in gesture naturalness and exhibits rapid convergence across languages, specifically Indonesian, Japanese, and Italian. Objective and subjective evaluations confirm improvements in communicative effectiveness. Notably, results reveal that individuals are more critical of gestures in their native language, expecting higher generative performance in familiar linguistic contexts. By releasing the TED-Culture Dataset, we facilitate future research on multilingual gesture generation for embodied agents. The study underscores the importance of cultural and linguistic adaptation in co-speech gesture synthesis, with implications for human-robot interaction design.

## 1 Introduction

As virtual agents and robots are becoming more popular, optimizing the interaction between humans and these technologies is becoming increasingly important. According to a human evaluation instrument, the ESI (Evaluation of Social Interaction) (Fisher and Griswold, 2010), some essential social skills are identified, such as approaching, speaking, turn-taking, gazing, and gesturing. These social interaction skills are equally applicable to the interaction between humans with virtual agents or robots. A significant portion of human interaction occurs through non-verbal means, frequently involving gestures made alongside spoken language (Knapp et al., 2013). Since the gestures contain rich non-verbal information, these movements play an important role in human communication (Studdert-Kennedy, 1994). The rise of telepresence in virtual/augmented reality, 3D animation, and social games highlights the importance of real-time gesture generation in dialogues and conversations (Lee et al., 2019a). Therefore, accompanying the natural co-speech gestures to virtual agents and robots is extremely desired.

Two main approaches to gesture generation are rule-based and data-driven methodologies (Liu et al., 2021). In addition, combining both approaches, hybrid systems have been introduced in some recent studies to generate natural and semantically meaningful gestures (Zhou et al., 2022). Rule-based systems can be repetitive and monotonous, while data-driven approaches leverage deep neural networks, from CNN (Habibie et al., 2021) to GANs (Goodfellow et al., 2014); (Liu et al., 2023). Despite GANs’ state-of-the-art performance, they face challenges like mode collapse and unstable training. Inspired by the success of the Stable Diffusion (Ho et al., 2020) in image creation, exploring its application in gesture generation is promising.

Gesture generation research is limited by data scarcity. Capturing finger motion accurately remains challenging. Studies have demonstrated a close relationship between speech and gestures across various cultures (Kita, 2009). However, existing models and datasets have not adequately addressed cultural or linguistic impacts on gesture generation. The BEAT Dataset (Liu H. et al., 2022) includes four languages and diverse cultural backgrounds but has limited speaker diversity and high data collection costs. Similarly, Gjaci et al. (2022) introduced a dataset featuring Indian and English speakers, however, both groups speak only in English, limiting its utility for investigating cultural factors. To address these gaps, a multimodal dataset that captures cultural differences, ensures speaker diversity, and includes detailed finger motion data would significantly advance the field.

Gesturing predominantly involves upper body and finger movements, with precise finger motion being particularly challenging. Improving finger motion quality could significantly enhance the authenticity and appropriateness of distal finger movements in social agents such as robots. Only two social robots, BERTI Bremner et al. (2009) and Erica Ishi et al. (2018), can render complete finger motions. Research on gesture generation for these robots is limited. Yoon et al. converted 2D poses to 3D poses and retargeted them to the NAO robot but did not address finger movements due to the absence of individual actuators of NAO’s fingers (Yoon et al., 2019). Therefore, the challenge of rendering finger motions on robots such as NAO using a dataset containing finger motion remains an unaddressed gap in the field.

To address the aforementioned challenges, our main contributions are as follows: (1) Introducing the TED-Culture Dataset for cross-cultural gesture generation based on linguistic cues; (2) Developing a novel generative model based on the DiffGesture framework (Zhu et al., 2023), achieving state-of-the-art performance on the TED-Expressive Dataset and rapid convergence across several languages in the TED-Culture Dataset; (3) Analyzing cultural factors using the TED-Culture Dataset, showing that people are more critical of outputs in familiar languages; and (4) Developing a robot prototype that maps our model’s gestures onto an NAO robot, enabling it to speak six languages with corresponding gestures.

## 2 Related works

### 2.1 Co-speech gesture generation

Data-driven approaches have gained popularity due to their ability to reduce manual efforts in designing rules, unlike rule-based methods. Nyatsanga et al. (2023) provide a comprehensive survey of these methods. Data-driven approaches are categorized into statistical and learning-based methods. Statistical approaches use models to derive rules from data, mapping input to gesture units for generation, as seen in Levine et al. (2010). With advancements in deep neural networks and increased human gesture data, end-to-end gesture generation systems have emerged. These systems include deterministic models like MLP (Kucherenko et al., 2020), CNN (Habibie et al., 2021), and RNN (Yoon et al., 2019); Yoon et al., 2020); (Liu X. et al., 2022), as well as non-deterministic models like VAEs (Ghorbani et al., 2023) and GANs (Yoon et al., 2020). Recent hybrid systems (Zhou et al., 2022) combine rule-based and data-driven methods to address issues like regression to the mean, showing superior performance. GAN-based systems have achieved cutting-edge results but face challenges like mode collapse and training instability. Inspired by Stable Diffusion (Ho et al., 2020) in image creation, new studies (Zhu et al., 2023) have adapted this framework for gesture generation, achieving state-of-the-art performance, though cross-cultural testing is still needed.

### 2.2 Human motion capture datasets

**FIGURE 1:**
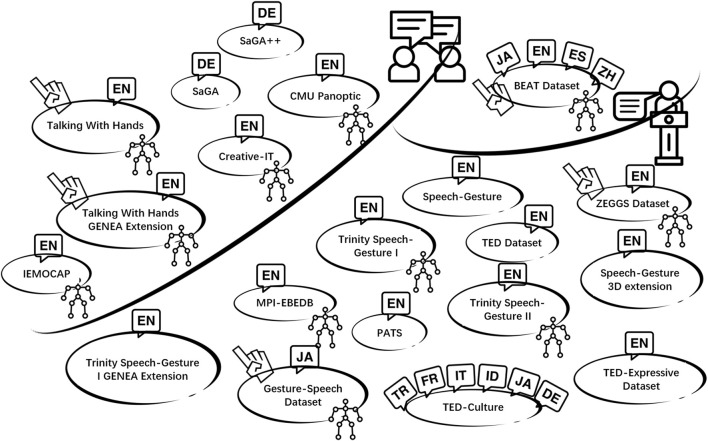
Summary of Datasets for Co-Speech Gesture Generation. Finger icons indicate datasets with high-quality finger motion, and motion capture icons denote those generated via optical motion capture. All datasets, except BEAT, are either monologues or conversations. Language icons represent dataset languages: EN (English), ES (Spanish), JA (Japanese), ZH (Chinese), DE (German), ID (Indonesian), TR (Turkish), FR (French), and IT (Italian).

Creating a dataset for human gesture motion involves two primary methods: optical motion capture (Takeuchi et al., 2017); (Lee et al., 2019b) and pose estimation from monocular videos (Yoon et al., 2019; Habibie et al., 2021). While the quality of optical motion capture is relatively higher than monocular video, it tends to yield smaller datasets due to its higher cost and the labor-intensive nature of the data collection. In contrast, pose estimation from monocular videos offers the advantage of generating larger datasets since a plethora of online videos are available for analysis. Despite the growing availability and sizes of multi-modal datasets, those encompassing high-quality finger motion remain scarce. Figure 1 provides an overview of datasets used for co-speech gesture generation. Five speech-gesture datasets incorporate high-quality finger motion, (namely (Takeuchi et al., 2017), (Lee et al., 2019b), (Yoon et al., 2022), (Ghorbani et al., 2023), and (Liu H. et al., 2022)), and they are all collected using optical motion capture devices. Among these, the Gesture-Speech Dataset (Takeuchi et al., 2017) and ZEGGS Dataset (Ghorbani et al., 2023) are presented in a monologue context, where only one person conducts an interview or talks and is then recorded. The Talking With Hands Dataset (Lee et al., 2019b), on the other hand, comprises multi-modal recordings of face-to-face spontaneous conversations involving two individuals, making it the largest motion capture and audio dataset for natural conversations to date. Other publicly available datasets lack high-quality finger motion, such as the TED Dataset (Yoon et al., 2019), which relies on pose estimation from monocular videos but is limited to 2D motion format, making it unsuitable for mapping onto social robots or 3D virtual agents and lacking finger motion data.

Fortunately, recent work from Habibie et al. (2021) has extended 2D skeletons into the three-dimensional realm. Yoon et al. (2020) expanded the TED Dataset from Yoon et al. (2019) by incorporating more TED videos into it, then converted all human poses into a 3D format using the 3D pose estimator (Pavllo et al., 2019). Additionally, Liu et al. introduced the TED-Expressive Dataset (Liu X. et al., 2022), addressing a limitation of the original TED Dataset Yoon et al. (2020) by including expressive co-speech finger movements alongside upper body key points. In the realm of culture-related datasets, the BEAT Dataset (Liu H. et al., 2022) places its main emphasis on capturing the emotions expressed by actors, and it offers multi-cultural and multi-language gesture data since the thirty participants are from ten countries. However, it is important to recognize that this dataset requires significant resources and has a limited variety of speaker identities, largely attributable to its reliance on optical motion capture technology. Consequently, among the existing 3D datasets, none have addressed the influence of culture or language on gesture generation while simultaneously ensuring efficient data collection and encompassing a diverse range of speaker identities.

## 3 TED-culture dataset

To address the scarcity of the culture-aware co-speech gesture dataset, we developed a new dataset called *TED-Culture Dataset*, featuring six different languages: Indonesian, Japanese, German, Italian, French, and Turkish. Inspired by the TED Dataset built by Yoon et al. (2019), Yoon et al. (2020), we choose the TEDx Talks channel on Youtube^1^ as the original source of our dataset. While TED Talks have certain limitations, such as the lack of representativeness of TED speakers in reflecting real-world diversity and the tendency of these professional and trained speakers to overuse gestures, the TED Talk video source has some advantages including ample data, diversity, and well-prepared gestures. We follow the dataset collection pipeline of Liu X. et al., (2022), excluding word-level alignments due to the use of auto-generated subtitles. Following the production process, we consolidated the individual language datasets into a unified “Merged” dataset.

Regarding the source videos of the TED-Culture Dataset, the average video length is 15 minutes. The total length of the source video is 60.1 h, with each language having a relatively even distribution of approximately 10 h each. The final dataset format represents 3D coordinates, encompassing multimodal aspects including Gesture, Audio, and Text. All poses are spine-centered, with 43 key points (13 upper body joints and 30 finger joints) defined in the dataset. The statistical information for the TED-Culture Dataset is presented in Table 1.

**TABLE 1:** Statistics information about the TED-Culture Dataset.

Language	Speakers	Valid clip number	Seconds	Hours
Turkish	21	306	11,729	3.3
French	29	457	9,701	2.7
Italian	35	580	11,651	3.2
Indonesian	29	351	8,343	2.3
German	39	901	14,142	3.9
Japanese	30	202	7,407	2.1
Total	183	2,797	62,974	17.5

In the table, we note a total of 183 speakers in our dataset, surpassing speaker counts in datasets captured by motion capture devices (typically involving fewer than or equal to 50 speakers), as well as most pose estimation datasets like Speech-Gesture (Ginosar et al., 2019) and Speech-Gesture 3D extension (Habibie et al., 2021). The TED-Culture Dataset comprises 2,797 valid clips, totaling 17.5 h with an average clip length of 23 s. The distribution of our dataset demonstrates a relatively even distribution across all languages. German holds the largest share at 22.3%, while Japanese has the smallest share at 12%. The distribution percentages for the other languages are as follows: Indonesian 13.1%, Italian 18.3%, French 15.4%, and Turkish 18.9%.

## 4 The proposed approach

**FIGURE 2:**
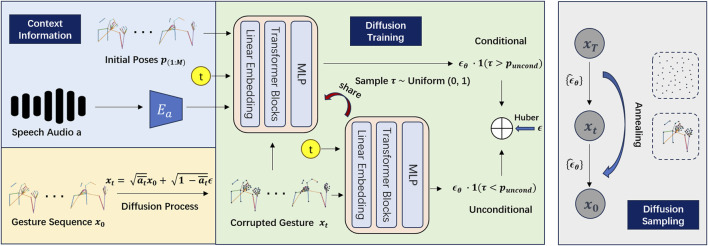
Overview of the DiffCulture Framework, based on the figure in Zhu et al. (2023).

Expanding upon insights from speech and its accompanying gestures, we have developed a deep learning approach focused on uncovering the intrinsic relationship between these modalities. Figure 2 provides an overview of our proposed *DiffCulture* framework, which is based on Zhu et al. (2023) and aims to enhance the fidelity of co-speech gesture generation. Unlike the original DiffGesture model (Zhu et al., 2023), our approach updates the objective function and modifies the architecture of the audio encoder. Despite testing other similar audio encoders in previous work (Zhu et al., 2023) (Zhi et al., 2023), no performance gains were observed. For details on the individual components of the framework, please refer to Zhu et al. (2023).

### 4.1 Problem formulation

For this gesture generation problem, we utilize the large-scale co-speech gesture training corpus introduced in Section 3, which focuses on videos featuring distinct and prominent upper body movements synchronized with speech to conduct the model learning process. Specifically, we assume every video clip has N frames, then we can define the co-speech audio sequence as $a=\left\{a_{1},\ldots ,a_{N}\right\}$ and annotate the per-frame human skeletons as $x=\left\{p_{1},\ldots ,p_{N}\right\}$ , where $p_{i}$ denotes the $i^{th}$ pose. These skeletal representations are pre-processed as the concatenation of unit direction vectors using the same method in the baselines as in Yoon et al. (2020); Liu X. et al. (2022). The unit direction vectors are represented as $p_{i}=\left[d_{i,1},d_{i,2}\ldots ,d_{i,J-1}\right]$ where $p_{i}$ means the pose description coordinates of the $i^{th}$ frame, J is the total joint number and $d_{i,j}$ stands for the $j^{th}$ unit direction vector among the J joints in the $i^{th}$ image frame. The diffusion model’s reverse denoising process G parameterized by θ is optimized to generate the human skeleton sequence x , taking into account the speech audio sequence a and initial poses $p_{1},.,p_{M}$ from the first M frames as conditioning factors. The primary objective of the framework is to generate a pose sequence that closely approximates the ground truth x . This objective can be mathematically formulated as $arg\;min_{\theta }\|x-G_{\theta }\left(a,p_{1},\ldots ,p_{M}\right)\|$ . In the context of deep learning, the system’s training phase involves providing a gesture sequence (x), an audio sequence (a), and seed poses (p) as inputs. In contrast, during the testing phase, only the audio sequence (a) and seed poses (p) are supplied, while the gesture sequence (x) is predicted.

### 4.2 Proposed model architecture

Figure 2 shows the DiffCulture Framework we developed, which is mostly based on the DiffGesture (Zhu et al., 2023). The orange area shows the forward diffusion process given the gesture sequence $x_{0}$ . After adding Gaussian noise to the gesture sequence $x_{0}$ based on the formulation $x_{t}=\sqrt{\bar{a_{t}}}x_{0}+\sqrt{1-\bar{a_{t}}}\epsilon$ , we will get a corrupted gesture $x_{t}$ . The blue area indicates the context information part, which includes the initial poses $p_{\left(1:M\right)}$ , speech audio a , and time embedding t . It is worth noting that we add another text modal to the context information when we do the ablation experiments. Then the given gesture sequence $x_{0}$ and context information were input into the green part together to indicate the conditional denoising process. The two neural network blocks consist of Linear Embedding, Transformer Blocks, and MLP, which receive multiple modalities in the context information and corrupted gestures with time embedding. Instead of the MSE loss utilized in Zhu et al. (2023), we use the Huber loss as our learning objective and do the element-wise plus conditional and unconditional embedding features together for further diffusion sampling. Huber loss is a robust loss function used in regression tasks that is less sensitive to outliers than the Mean Squared Error (MSE) loss. Given that we employ the Huber loss as our learning objective, we can simplify the training objective through parameterization to an ensemble of Huber loss formulated as:

$$l_{n}=\left\{\begin{array}{cc} 0.5{\left(x_{n}-y_{n}\right)}^{2},\; & \text{if }|x_{n}-y_{n}|<delta\\ delta\left(|x_{n}-y_{n}|-0.5\;delta\right),\; & otherwise \end{array}\right.$$ (1)

where ${\left(x_{n}-y_{n}\right)}^{2}=L\left(\theta \right)=E_{q}\left[\|\epsilon -\epsilon_{\theta }\left(\sqrt{\bar{a_{t}}}x_{0}+\sqrt{1-\bar{a_{t}}}\epsilon ,c,t\right){\|}^{2}\right]$ . Here t is uniformly chosen from 1 to T . As we concurrently train the model in both conditional and unconditional settings, a trainable masked embedding with probability $p_{\mathrm{uncond}}$ replaces the context c , and the diffusion model predicts the noise in the unconditional settings.

Lastly, the grey area highlights the diffusion sampling phase, where we introduce the Diffusion Gesture Stabilizer, which employs an annealed noise sampling strategy to address temporal inconsistencies. Additionally, to integrate implicit classifier-free guidance, we jointly train conditional $\left(1-p_{\mathrm{uncond}}\right)$ and unconditional $\left(p_{\mathrm{uncond}}\right)$ models. This approach enables us to balance between diversity and quality when performing inference.

## 5 Experiments

### 5.1 Co-speech gesture datasets

#### 5.1.1 TED-expressive

In contrast to the TED Dataset (Yoon et al., 2019; Yoon et al., 2020), which includes only 10 upper body key points and lacks detailed finger movements, the TED-Expressive Dataset (Liu X. et al., 2022) offers a more comprehensive representation of both finger and body movements. This enhancement is achieved using the state-of-the-art 3D pose estimator ExPose (Choutas et al., 2020), which captures detailed pose information. Consequently, the TED-Expressive Dataset annotates the 3D coordinates of 43 key points, encompassing 13 upper body joints and 30 finger joints.

#### 5.1.2 TED-culture

The data collection pipeline for the TED-Culture Dataset follows the same methodology as the TED-Expressive Dataset, resulting in an identical representation format. In this work, we focus on the TED-Culture Merged dataset, with experimental results for specific languages provided in the Project Website^2^.

### 5.2 Experimental settings

#### 5.2.1 Baselines

We evaluate our method on two benchmark datasets, comparing it with several state-of-the-art methods developed in recent years: 1) Attention Seq2Seq (Yoon et al., 2019) elaborates on the attention mechanism to generate pose sequences from speech text; 2) Speech2Gesture (Ginosar et al., 2019) employs speech audio spectrums as input to adversarially generate speech gestures; 3) Joint Embedding (Ahuja and Morency, 2019) maps text and motion to the same embedding space to generate outputs from motion description text; 4) Trimodal (Yoon et al., 2020) serves as a robust baseline that learns from text, audio, and speaker identity to generate gestures, significantly outperforming previous methods; 5) HA2G (Liu X. et al., 2022) introduces a hierarchical audio learner that captures information across different semantic granularities, surpassing former methods; and 6) DiffGesture (Zhu et al., 2023) leverages the stable diffusion model (Ho et al., 2020) and Transformer architecture (Vaswani et al., 2017), achieving state-of-the-art performance. We also present evaluations directly on the pseudo Ground Truth annotated in the dataset.

#### 5.2.2 Implementation details

In our experiments, we utilize two datasets: TED-Expressive (Liu X. et al., 2022) and TED-Culture. We preprocess these datasets following the method outlined in Yoon et al. (2020), setting the length of each pose sequence N to 34 frames. Additionally, the length of the seed gesture is set to M=4 , representing the gestures of the first four frames used for reference during inference. To eliminate the effect of joint lengths and root motion, we follow (Yoon et al., 2019) and represent joint positions using J−1 3D directional unit vectors. For audio processing, we employ an audio encoder consisting of three convolutional layers, each followed by a Rectified Linear Unit (ReLU) activation layer and ending with a one-dimensional AdaptiveAvgPool layer. This configuration extracts features directly from raw audio clips, encoding them into N audio feature vectors, each with 32 dimensions. These audio features are concatenated with the initial poses to form the conditional context for the diffusion model. In the diffusion process, we use T=500 timesteps, with variances increasing linearly from $\beta_{1}=1\times 10^{-4}$ to $\beta_{T}=0.02$ . However, for specific cases like the Japanese dataset, additional experiments were conducted with increased timesteps of 1,000 and 1,500 to compare their effects as experimental results indicate that 500 epochs are insufficient for the model to converge fully on these datasets. For the Stabilizer (Zhu et al., 2023), $t_{0}$ can be adjusted between 20 and 30 for thresholding, and a quadratic non-increasing function $\sigma_{a}\left(t\right)$ is applied for smooth sampling. The hidden dimension of the transformer blocks is set to 512 for both the TED-Expressive and TED-Culture. We use eight transformer blocks, each comprising a multi-head self-attention block and a feed-forward network. The Adam optimizer is used with a learning rate of $5\times 10^{-4}$ . The threshold for the Huber loss is set to 0.1. Training the model using each separate language dataset TED-Culture takes approximately 1 h (6 h for Merged) and 44 h for TED-Expressive on a single NVIDIA A100 Tensor Core GPU on HPC.

### 5.3 Quantitative evaluation

For quantitative analysis, we employ evaluation metrics previously used in co-speech gesture generation (Liu X. et al. 2022); Zhu et al., 2023) and related tasks such as music-to-dance (Sun et al., 2020).

#### 5.3.1 Quantitative metrics

##### 5.3.1.1 Fréchet gesture distance (FGD)

Like the commonly used Fréchet Inception Distance (FID) metric in image generation research, the FGD metric serves the purpose of quantifying the dissimilarity between the distribution of synthesized gestures and that of real data. Yoon et al. (2020) introduce the FGD metric by training a skeleton sequence auto-encoder to extract features from both real gesture sequences X and generated gesture sequences

$$\hat{X}:FGD\left(X,\hat{X}\right)=\|\mu_{r}-\mu_{g}{\|}^{2}+T_{r}(\sum_{r}+\sum_{g}-2{\left(\sum_{r}\sum_{g}\right)}^{1/2}),$$

where $\mu_{r}$ and $\sum_{r}$ represent the first and second moments of the latent feature distribution of the real gestures X , while $\mu_{g}$ and $\sum_{g}$ correspond to the first and second moments of the latent feature distribution of the generated gestures $\hat{X}$ .

##### 5.3.1.2 Beat Consistency Score (BC)

The Beat Consistency Score (BC) Li et al. (2022), Li et al. (2021) is designed to gauge the correlation between motion and speech beats. Recognizing the diversity in kinematic velocities among different joints, Liu X. et al. (2022) propose employing changes in the included angle between bones to identify motion beats. To initiate this process, they compute the Mean Absolute Angle Change (MAAC) for angle $\theta_{i}$ between consecutive frames using the following equation:

$$MAAC\left(\theta_{j}\right)=\frac{\sum_{s=1}^{S}\sum_{t=1}^{T-1}\|\theta_{j,s,t+1}-\theta_{j,s,t}{\|}_{1}}{S*\left(T-1\right)},$$

where S represents the total number of clips within the dataset, T signifies the number of frames contained in each clip, and $\theta_{j,s,t}$ corresponds to the included angle between the $j^{t}h$ and ${\left(j+1\right)}^{t}h$ bone of the $s^{t}h$ clip at time-step t . The angle change rate for frame t within the $s^{t}h$ clip can be computed as

$$\frac{1}{J-1}\sum_{j=1}^{J-1}(\|\theta_{j,s,t+1}-\theta_{j,s,t}{\|}_{1}/MAAC\left(\theta_{j}\right)),$$

Subsequently, kinematic beats are identified as local optima whose first-order difference exceeds a predefined threshold. To detect audio beats, we follow the methodology outlined in Li et al. (2022), utilizing the onset strength (Ellis, 2007). The Beat Consistency score is then determined as the average distance between each audio beat and its closest motion beat:

$$BC=\frac{1}{n}\sum_{i=1}^{n}\exp(-\frac{\min\forall t_{j}^{x}\in B^{x}\|t_{i}^{x}-t_{j}^{y}{\|}^{2}}{2\sigma^{2}}),$$

where $t_{i}^{x}$ represents the i -th audio beats, $B^{y}=t_{i}^{y}$ denotes the set of kinematic beats, and σ is a parameter used for sequence normalization.

##### 5.3.1.3 Diversity

This metric assesses the disparities in generated gestures that correspond to different inputs, as detailed in Lee H.-Y. et al. (2019). When calculating FGD, we employ the same feature extractor to map synthesized gestures into latent feature vectors and determine the mean feature distance. Specifically, in some studies (Liu X. et al., 2022); (Zhu et al., 2023) 500 randomly selected generated samples are used to compute the mean absolute error between the features and shuffled features.

#### 5.3.2 Evaluation results

Table 2 presents the objective evaluation results for the TED-Expressive and TED-Culture Merged datasets. Baseline results for TED-Expressive are sourced from Zhu et al. (2023). Our model, DiffCulture, surpasses all baselines and achieves state-of-the-art performance on the TED-Expressive Dataset. Although the performance improvement over DiffGesture is minimal, DiffCulture still demonstrates superior overall results. Specifically, for the BC and Diversity metrics, our model, despite being slightly weaker than DiffGesture, outperforms all other models. On the TED-Culture Merged dataset, DiffGesture achieves the best FGD score, improving by nearly 30% compared to HA2G and DiffCulture. Additionally, since BC and Diversity measure motion-audio beat correlation and variation, these metrics for Ground Truth should not be treated as upper bounds. Notably, our results are on par with Ground Truth, indicating high-quality generated gestures. Results for specific languages in the TED-Culture Dataset are listed in the Project Website^3^, demonstrating both SOTA performance and faster convergence across languages compared with Zhu et al., (2023), especially in Indonesian, Japanese, and Italian. It is worth noting that during the evaluation process, the BC and Diversity metrics exhibit significant fluctuations, highlighting the need for further refinement of the quantitative metrics.

**TABLE 2:** The Quantitative Results on TED-Expressive Liu X. et al. (2022) and TED-Culture Merged.

	TED-expressive Liu et al. (2022b)			TED-culture		
Methods	FGD ↓	BC ↑	Diversity ↑	FGD ↓	BC ↑	Diversity ↑
Ground Truth	0	0.703	178.827	0	0.702	181.900
Attention Seq2Seq (Yoon et al., 2019)	54.920	0.152	122.693	27.858	0.205	150.985
Speech2Gesture (Ginosar et al., 2019)	54.650	0.679	142.489	53.676	0.567	136.512
Joint Embedding (Ahuja and Morency, 2019)	64.555	0.130	120.627	52.993	0.135	120.380
Trimodal (Yoon et al. 2020)	12.613	0.563	154.088	12.026	0.396	146.988
HA2G (Liu et al., 2022b)	5.306	0.641	173.899	5.919	0.310	160.225
DiffGesture (Zhu et al., 2023)	2.600	**0.718**	**182.757**	**4.216**	**0.728**	**175.025**
DiffCulture (Ours)	**2.398**	0.715	177.814	5.532	0.722	160.603

We compare the proposed diffusion-based method against recent state-of-the-art (SOTA) methods (Yoon et al., 2019); (Ginosar et al., 2019); (Ahuja and Morency, 2019); (Yoon et al., 2020); (Liu X. et al., 2022); (Zhu et al., 2023) and ground truth. Lower values are better for FGD, while higher values are better for the other metrics.

Bold values denote the best performance for each respective metric in the table.

### 5.4 User evaluation

**FIGURE 3:**
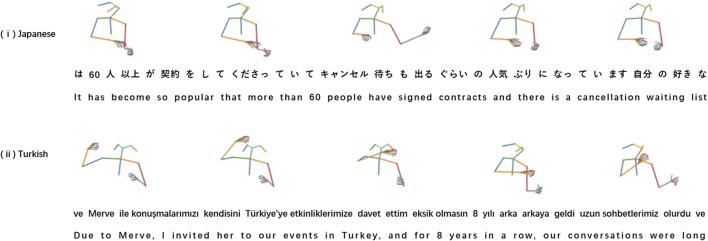
Visualization Results with subtitles of our DiffCulture model on the TED-Culture Dataset. Two cases are selected: (i) a Japanese case and (ii) a Turkish case. Both cases are visualized with corresponding subtitles, with both the original language and translation format in English. Note that the stride between every frame is 20.

Given that the generated gestures will ultimately be used in interactions with virtual agents or social robots, involving real individuals or users in the evaluation process is the ideal approach for assessing the quality and effectiveness of these gestures. We will analyze the result from the cultural perspective since the particularity of our dataset is multilingual and multicultural. Figure 3 displays two cases, one in Japanese and the other in Turkish. It is noteworthy that translation is subject to a phenomenon known as the “word order change phenomenon”, wherein the order of morphemes (words or parts of words) in the target language differs from that in the source language after translation. Consequently, the word order in the English translation may not directly correspond to the order of the gesture sequence due to changes in the timing of morphemes. Take the Japanese case illustrated in the figure as an example: in the Japanese sentence, “60” is positioned at the beginning, whereas in the English sentence, “60” appears in the middle. When interpreting semantic information conveyed through gestures, the focus should be on the original subtitles rather than the translated version. The translated version is intended to facilitate understanding for individuals who do not comprehend the original language but understand English. For example, in the Japanese case, the framework generates iconic gestures depicting “become so popular”, constituting the third key frame where the gesture extends the left arm to indicate emphasis. In the Turkish case, a similar phenomenon is observed with phrases like “and for 8 years in a row” and “our conversations were long”, corresponding to the fourth and fifth key frames in the figure.

#### 5.4.1 Case study

To better validate the qualitative performance, we conducted a user case study on the generated co-speech gestures. The study involved 42 participants aged between 18 and 45 years, with 11 men, 30 women, and 1 non-binary person. From the language perspective, English was the predominant language spoken among participants, with 17 individuals, followed closely by Chinese and Indonesian, spoken by 14 and 7 individuals, respectively. The remaining 4 participants spoke Vietnamese or other less common languages. Each participant was required to assess the quality and coherence of the motion, with all clips presented without labels. A total of 136 cases^4^ were selected, comprising 17 (3 for Indonesian, 2 for Japanese, 3 for German, 3 for Italian, 3 for French, and 3 for Turkish) for each baseline (seven different frameworks and one ground truth). When distributing the questionnaires offline via campus bulletin boards or online through email, we use Qualtrics’ randomization function to select three testing videos for each method, resulting in a total of 24 videos per questionnaire. The Mean Opinion Scores (MOS) rating protocol was adopted, where each participant rated three aspects of the generated motions: naturalness, smoothness, and synchrony with speech. The results are presented in Table 3. Interestingly, the Attention Seq2Seq model achieved the highest subjective evaluation scores, in contrast to the results obtained in objective evaluation, where the Attention Seq2Seq framework performed worse. Qualitative analysis revealed that gestures generated by this model exhibited a slow and rigid behavior, which intuitively might not lead to high subjective evaluation scores. However, the slower gestures made by this model contributed to smoother and more coherent movements for virtual agents driven by gestures. In contrast, other models such as DiffGesture were perceived to produce overly jerky gestures due to pronounced changes between each frame, leading to less favorable and poorer subjective evaluation results. Overall, our DiffCulture model performed intermediately compared to the other models, surpassing the Ground Truth and some of the other models such as HA2G.

**TABLE 3:** User Study Results.

Methods	Naturalness	Smoothness	Synchrony
Ground Truth	2.21 (0.94)	1.96 (0.74)	2.21 (0.86)
Attention Seq2Seq (Yoon et al., 2019)	**3.15** (0.69)	**3.57** (0.52)	**3.05** (0.64)
Speech2Gesture (Ginosar et al., 2019)	2.91 (0.73)	2.56 (0.65)	3.03 (0.80)
Joint Embedding (Ahuja and Morency, 2019)	2.66 (0.65)	3.37 (0.44)	2.41 (0.64)
Trimodal (Yoon et al., 2020)	2.74 (1.01)	2.27 (0.82)	2.74 (1.04)
HA2G (Liu et al., 2022b)	1.98 (0.69)	1.86 (0.64)	1.91 (0.64)
DiffGesture (Zhu et al. 2023)	2.29 (0.81)	2.11 (0.59)	2.43 (0.63)
DiffCulture (Ours)	2.59 (0.83)	2.27 (0.70)	2.52 (0.80)

The ratings for motion naturalness, smoothness, and synchrony are assessed on a scale of 1–5, where 5 indicates the highest performance. All the results in the table are presented in the format of Average (SD).

Bold values denote the best performance for each respective metric in the table.

From a language acquisition perspective, we exclusively examine the subjective evaluation results from seven participants who are proficient in Indonesian as either their first or second language. The study includes two conditions: one focusing on subjective evaluation results for Indonesian videos and the other for non-Indonesian videos. Table 4 presents the perception of Indonesian participants in these two scenarios. According to the table, participants assigned similar scores for smoothness to videos in both familiar and unfamiliar languages. However, participants tended to assign lower scores to gestures performed in their native language, with Indonesian videos receiving significantly lower scores in naturalness and synchrony compared to non-Indonesian videos. This indicates that individuals are more critical of co-speech gestures in their native language and expect higher performance from generative models.

**TABLE 4:** The correlation between language acquisition and the subjective perception of the participants.

Video Type	Naturalness	Smoothness	Synchrony
Indonesian Videos	1.94 (1.07)	**2.18** (1.16)	1.80 (1.16)
Non-Indonesian Videos	**2.34** (1.05)	**2.18** (1.14)	**2.34** (1.01)

Bold values denote the best performance for each respective metric in the table.

### 5.5 Ablation studies

We conducted text-embedding experiments to investigate whether using word embeddings from different languages would influence the training process and results. Unlike the typical ablation study that usually tests how system performance changes when components are removed, we extended the proposed framework to include text modality handling and performed three sets of experiments by varying the language of the FastText embeddings, using TED-Culture French. Aside from the language of the FastText embeddings, we also adjusted the number of training epochs, increasing them from 500 to 1,000 when using the French FastText to embed the subtitles. Table 5 presents the results of these ablation experiments on text embedding. From the table, it is evident that the English FastText Word Embedding outperforms the other two conditions, even though in the last case we trained the model for 1,000 epochs to ensure complete convergence. Conversely, when using French FastText Word Embedding, the results indicate that 500 epochs are insufficient for the framework with text modality to converge to an optimal state. Even with 1,000 epochs, the model using French FastText still performs worse than its English counterpart, despite the longer training time. We can conclude that the language of word embeddings based on FastText does not significantly impact the final performance of the framework, but it can affect the convergence time.

**TABLE 5:** Ablation study on the impact of using corresponding text embeddings in the DiffCulture model after incorporating the text modality on TED-Culture French.

Methods	FGD ↓	BC ↑	Diversity ↑
Without Text Modality, 500	5.053	0.747	91.817
English Text Embedding, 500	**5.003**	0.749	96.225
French Text Embedding, 500	10.691	**0.750**	**101.947**
French Text Embedding, 1,000	5.205	0.745	96.936

Bold values denote the best performance for each respective metric in the table.

## 6 Robot prototype

Unlike mapping gestures to virtual agents, some constraints exist when mapping gestures to robots because the joints of the robots are motor-driven and not as flexible as those of virtual agents. Since the output of our framework consists of 3D directional vectors, we first need to calculate the radian values for each vector. These radian values often exceed the joints’ angle range, making post-processing an essential step. We applied different post-processing methods to the different joints to make it look as natural as possible and consistent with the gesture motion trends generated by the model. To mitigate the jittering issue in the generated gestures, Bézier interpolation is employed during the retargeting process. Since the robot has difficulty walking and the dataset focuses only on the upper body, not all joints are used in this project. We concentrate on the head and arms, as well as hands which only have open and close functions. Specifically, we utilize 12 degrees of freedom (DoF) in the upper body, namely HeadYaw, HeadPitch, RShoulderPitch, RShoulderRoll, RElbowYaw, RElbowRoll, RWristYaw, LShoulderPitch, LShoulderRoll, LElbowYaw, LElbowRoll, and LWristYaw, plus the open and close functions of the hands. All the robot prototype codes are available at this repository^5^, and the playlist^6^ includes both the outputs of the generative model visualized as skeletons and the robot prototype demonstrations for better comparison.

## 7 Conclusion

In this paper, we present a large-scale monologue dataset for cross-cultural gesture generation grounded in language and refine the gesture generation model based on Zhu et al. (2023). Additionally, we devise a state-of-the-art co-speech gesture framework and implement the generated gestures on the NAO robot, enabling synchronized speech and gesture performance. We conduct culturally subjective evaluations and an ablation study, validating the cultural relevance of co-speech gestures and demonstrating that word embeddings may not need to correspond to the language of the text. However, certain limitations are evident. Firstly, low subjective evaluation scores for Ground Truth indicate the dataset’s relative lack of quality. Additionally, during the dataset creation process, the filtering of valid clips lacks manual filtering. Furthermore, the experiments were conducted exclusively on the NAO robot, which has limited or no finger mobility, thus restricting the generalizability of the findings. To address this, future work could explore testing on more advanced platforms with greater dexterity to validate and extend the results.

## Data Availability

The datasets presented in this study can be found in online repositories. The names of the repository/repositories and accession number(s) can be found in the article/Supplementary Material.

## Footnotes

1
https://www.youtube.com/@TEDx

2
https://yixin-shen-1218.github.io/TED_Culture

3
https://yixin-shen-1218.github.io/TED_Culture

4
https://www.youtube.com/playlist?list=PLTTEUXLjZvB9-ViI1ZkmpZdphsQb92cAt

5
https://github.com/Yixin-Shen-1218/NAO_Gesture_Generation

6
https://www.youtube.com/playlist?list=PLTTEUXLjZvB8qEKfwHjQIgOJ3ahP_gOPS
